# Absolute Electronic Energetics and Quantitative Work Functions of Liquids from Photoelectron Spectroscopy

**DOI:** 10.1021/acs.accounts.2c00548

**Published:** 2023-01-04

**Authors:** Bernd Winter, Stephan Thürmer, Iain Wilkinson

**Affiliations:** † Molecular Physics Department, Fritz-Haber-Institut der Max-Planck-Gesellschaft, Faradayweg 4-6, 14195 Berlin, Germany; ‡ Department of Chemistry, Graduate School of Science, Kyoto University, Kitashirakawa-Oiwakecho, Sakyo-Ku, Kyoto 606-8502, Japan; § Institute of Electronic Structure Dynamics, Helmholtz-Zentrum Berlin für Materialien und Energie, Hahn-Meitner-Platz 1, 14109 Berlin, Germany

## Abstract

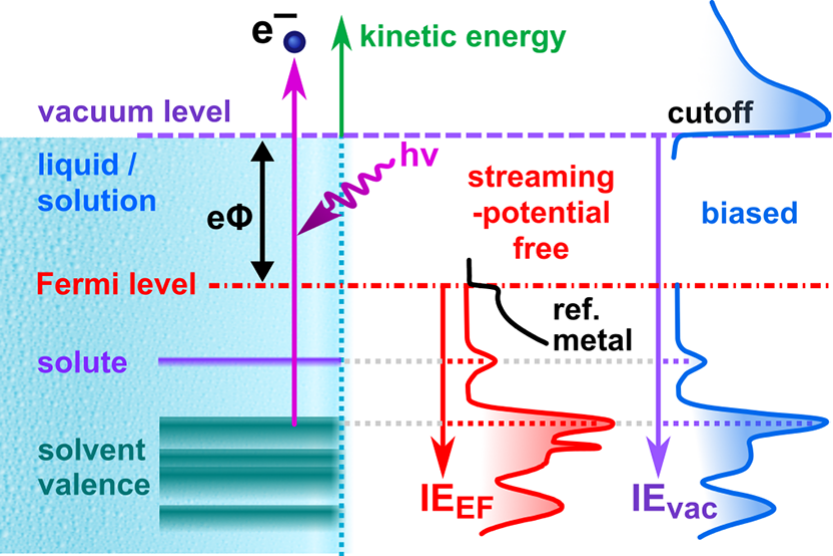

Liquid-jet photoelectron spectroscopy (LJ-PES) enabled a breakthrough in the experimental study of the electronic structure of liquid water, aqueous solutions, and volatile liquids more generally. The novelty of this technique, dating back over 25 years, lies in stabilizing a continuous, micron-diameter LJ in a vacuum environment to enable PES studies. A key quantity in PES is the most probable energy associated with vertical promotion of an electron into vacuum: the vertical ionization energy, VIE, for neutrals and cations, or vertical detachment energy, VDE, for anions. These quantities can be used to identify species, their chemical states and bonding environments, and their structural properties in solution. The ability to accurately measure VIEs and VDEs is correspondingly crucial. An associated principal challenge is the determination of these quantities with respect to well-defined energy references. Only with recently developed methods are such measurements routinely and generally viable for liquids. Practically, these methods involve the application of condensed-matter concepts to the acquisition of photoelectron (PE) spectra from liquid samples, rather than solely relying on molecular-physics treatments that have been commonly implemented since the first LJ-PES experiments. This includes explicit consideration of the traversal of electrons to and through the liquid’s surface, prior to free-electron detection. Our approach to measuring VIEs and VDEs with respect to the liquid vacuum level specifically involves detecting the lowest-energy electrons emitted from the sample, which have barely enough energy to surmount the surface potential and accumulate in the low-energy tail of the liquid-phase spectrum. By applying a sufficient bias potential to the liquid sample, this low-energy spectral tail can generally be exposed, with its sharp, low-energy cutoff revealing the genuine kinetic-energy-zero in a measured spectrum, independent of any perturbing intrinsic or extrinsic potentials in the experiment. Together with a precisely known ionizing photon energy, this feature enables the straightforward determination of VIEs or VDEs, with respect to the liquid-phase vacuum level, from any PE feature of interest. Furthermore, by additionally determining solution-phase VIEs and VDEs with respect to the common equilibrated energy level in condensed matter, the Fermi level—the generally implemented reference energy in solid-state PES—solution work functions, eΦ, and liquid-vacuum surface dipole effects can be quantified. With LJs, the Fermi level can only be properly accessed by controlling unwanted surface charging and all other extrinsic potentials, which lead to energy shifts of all PE features and preclude access to accurate electronic energetics. More specifically, conditions must be engineered to minimize all undesirable potentials, while maintaining the equilibrated, intrinsic (contact) potential difference between the sample and apparatus. The establishment of these liquid-phase, accurate energy-referencing protocols importantly enables VIE and VDE determinations from near-arbitrary solutions and the quantitative distinction between bulk electronic structure and interfacial effects. We will review and exemplify these protocols for liquid water and several exemplary aqueous solutions here, with a focus on the lowest-ionization- or lowest-detachment-energy PE peaks, which importantly relate to the oxidative stabilities of aqueous-phase species.

## Key References

- ThürmerS.; MalerzS.; TrinterF.; HergenhahnU.; LeeC.; NeumarkD. M.; MeijerG.; WinterB.; WilkinsonI.Accurate Vertical Ionization Energy and Work Function Determinations of Liquid Water and Aqueous Solutions. Chem. Sci.2021, 12, 10558–10582.3444755010.1039/d1sc01908bPMC8356740(1)*An extended discussion of accurate and general energy referencing in LJ-PES measurements and an exploration of the VIEs and work functions of liquid water and select aqueous solutions. The basics from this study are summarized in this account*.
- CredidioB.; PuginiM.; MalerzS.; TrinterF.; HergenhahnU.; WilkinsonI.; ThürmerS.; WinterB.Quantitative electronic structure and work-function changes of liquid water induced by solute. Phys. Chem. Chem. Phys.2022, 24, 1310–1325.3460489510.1039/d1cp03165aPMC8768487(2)*Concentration-dependent LJ-PES measurements of an electrolyte and a surfactant aqueous solution, where IE variations of solvent and solute features were accurately tracked. Data analysis revealed distinct differences in behavior, allowing solution work function and bulk electronic-structure changes to be differentiated*.
- MalerzS.; TrinterF.; HergenhahnU.; GhristA.; AliH.; NicolasC.; SaakC.-M.; RichterC.; HartwegS.; NahonL.; LeeC.; GoyC.; NeumarkD. M.; MeijerG.; WilkinsonI.; WinterB.; ThürmerS.Low-energy constraints on photoelectron spectra measured from liquid water and aqueous solutions. Phys. Chem. Chem. Phys.2021, 23, 8246–8260.3371021610.1039/d1cp00430a(3)*Determination of low-eKE thresholds for accurate determinations of liquid water and aqueous solution IEs using LJ-PES. A complete deterioration of native peak profiles was observed at eKEs below 10–13 eV, preventing accurate extraction of native IE values following UV ionization*.

## Introduction

As first reported in 1997,^4^ the photoelectrons (PEs) emitted from volatile liquid microjets (LJs) can be detected without scattering from the gas-phase molecules that naturally evaporate from and surround them, providing direct access to bound electron energetics in liquids. With subsequent developments of modern spectrometers with more sophisticated differential pumping schemes, the requirements for μm-sized LJs were partially mitigated. This allowed larger-scale liquid-phase samples, including so-called flat liquid jets and moist solids, to be studied with higher electron collection efficiencies and potentially under near-ambient-pressure conditions.^5−7^ Valence and core-level PE spectra of both solvent and solute species can correspondingly be directly measured. Core-level measurements offer exceptional element and local-environment sensitivity, as recently reviewed.^5,8,9^ In contrast, valence PE spectra are typically neither element- nor site-specific and are arguably more difficult to interpret. Thus, valence studies have mainly focused on easily identifiable solute features, such as the highest-electron-kinetic-energy (eKE) peaks recorded from simple electrolytes and transition-metal complexes.^10^ Generally in LJ photoelectron spectroscopy (PES), liquid-phase ionization energies (IEs) and detachment energies (DEs)^11^ or, mutually equivalently, electron binding energies (eBEs) are the primary quantities of interest. These quantities correspond to the energies required to release (photo)electrons into vacuum and are specifically termed vertical IEs (VIEs) or DEs (VDEs)^11^ when the electrons are liberated without concurrent geometric structural rearrangement. Such quantities are determined from the energetic positions of maximum intensity of discrete PE peaks and importantly identify a species, its chemical state, bonding environment, and structural properties in solution.

**Figure 1:**
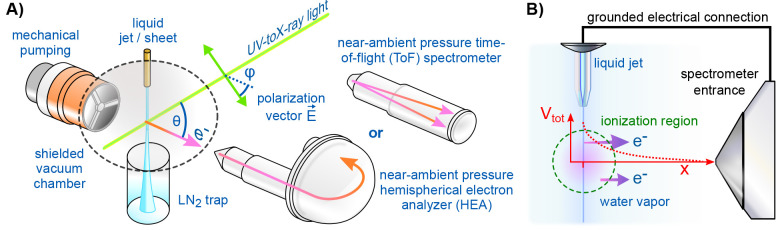
(A) Sketch of the essential components of a LJ-PES experiment. (B) Expanded view of the ionizing-light-sample-spectrometer interaction region. The light spot (dashed green circle) ionizes the LJ and surrounding gas. A potential gradient, *V*_tot_, between the sample and analyzer leads to different average photoelectron accelerations from the liquid and vapor phases (purple arrows).

Figure 1A sketches the essential parts of an LJ-PES experiment. The LJ or liquid sheet/flatjet^12,13^ is injected into vacuum via an appropriate nozzle, held and fine-adjusted using a μm-precision XYZ-manipulator within an electrically and magnetically shielded vacuum chamber. The laminar-phase of the jet is photoionized, and the PEs are detected by an electron spectrometer, with its entrance aperture positioned a few hundred micrometers from the LJ surface. Typically, hemispherical electron analyzers (HEAs) or magnetic-bottle (MB) or field-free time-of-flight spectrometers (ToFs) are utilized (see ref (14)). HEAs are commonly used in conjunction with soft-X-ray (synchrotron) radiation to measure eKEs up to 1000 eV with high energy resolution. MB-ToFs offer high electron collection efficiencies and have traditionally been applied in lower-eKE studies, often in conjunction with UV or EUV laser ionization. The liquid sample is usually frozen out by a liquid nitrogen (LN_2_)-cooled trap, placed opposite the injection point within the vacuum chamber, but specialized catcher units can alternatively be used to extract the LJ through a small orifice.^15−17^ The LJ-chamber vacuum conditions are typically maintained in the 10^–3^–10^–5^ mbar range, depending on the type of jet and LJ collector used, using suitable combinations of high-throughput mechanical pumping (turbomolecular pumps), LN_2_-cooled cold traps, and potentially a LJ catcher unit. Ionizing-light introduction and PE extraction are generally achieved through differential pumping stages.

Figure 1B depicts a LJ in front of an electron-detector entrance aperture and highlights important but often inadmissibly neglected aspects of LJ-PES: Surface charging and work function, eΦ, differences between the LJ and analyzer orifice (i.e., the common ground of the whole system) inevitably generate electric potentials between the sample and detector. The PEs experience different field strengths due to such parasitic potentials, labeled *V*_tot_ in the figure, leading to extrinsic energetic shifts between the PE features and making the accurate measurement of electronic energetics—a primary topic in this Account—highly challenging. Particularly, electrons originating from gas-phase molecules experience a different field strength, depending on their position of birth. This leads to a broadening of the gas-phase PE features and differential energetic shifts of the gas- and liquid-phase peaks, rendering the former an unreliable liquid-phase energy reference. A unique and complicating issue with LJs is electrokinetic charging,^18−20^ resulting from the disruption of an electric double layer between the flowing liquid and inner wall of the capillary or pinhole^21^ that forms the in-vacuum LJ. This leads to the so-called streaming potential, Φ_str_, which plays a particularly important role when one aims to quantitatively determine a solution work function. Additional sources of parasitic jet-surface charging and their implications will be detailed below.^22^

## Interpretation of Liquid-Phase Photoelectron Spectra

The measured quantity in a PES experiment is the eKE. In the case of direct, primary photoemission, this can be used, along with a known photon energy, ℏω, to calculate electron IEs, via IE = ℏω – eKE, assuming the eKE has not been altered, e.g., by unwanted inelastic scattering or electric fields in the experiment.^22^ Specifically considering PE peak maxima, the VIEs calculated in this way reveal the energies required to liberate electrons from bound states within the sample and place them “just outside” of it and into vacuum, as detailed later. Such considerations and calculations are generally straightforward in the gas-phase. However, additional complications arise with condensed matter, where electrons cross from a bulk phase into vacuum, via a surface, before being detected. A different interpretation of the IE correspondingly emerges in condensed-phase PES, with an alternative energy-reference point usually being implemented, as introduced below. Before discussing such interpretations and practices in detail, however, we will briefly outline some further complexities encountered in liquid-phase PES.^1^

**Figure 2:**
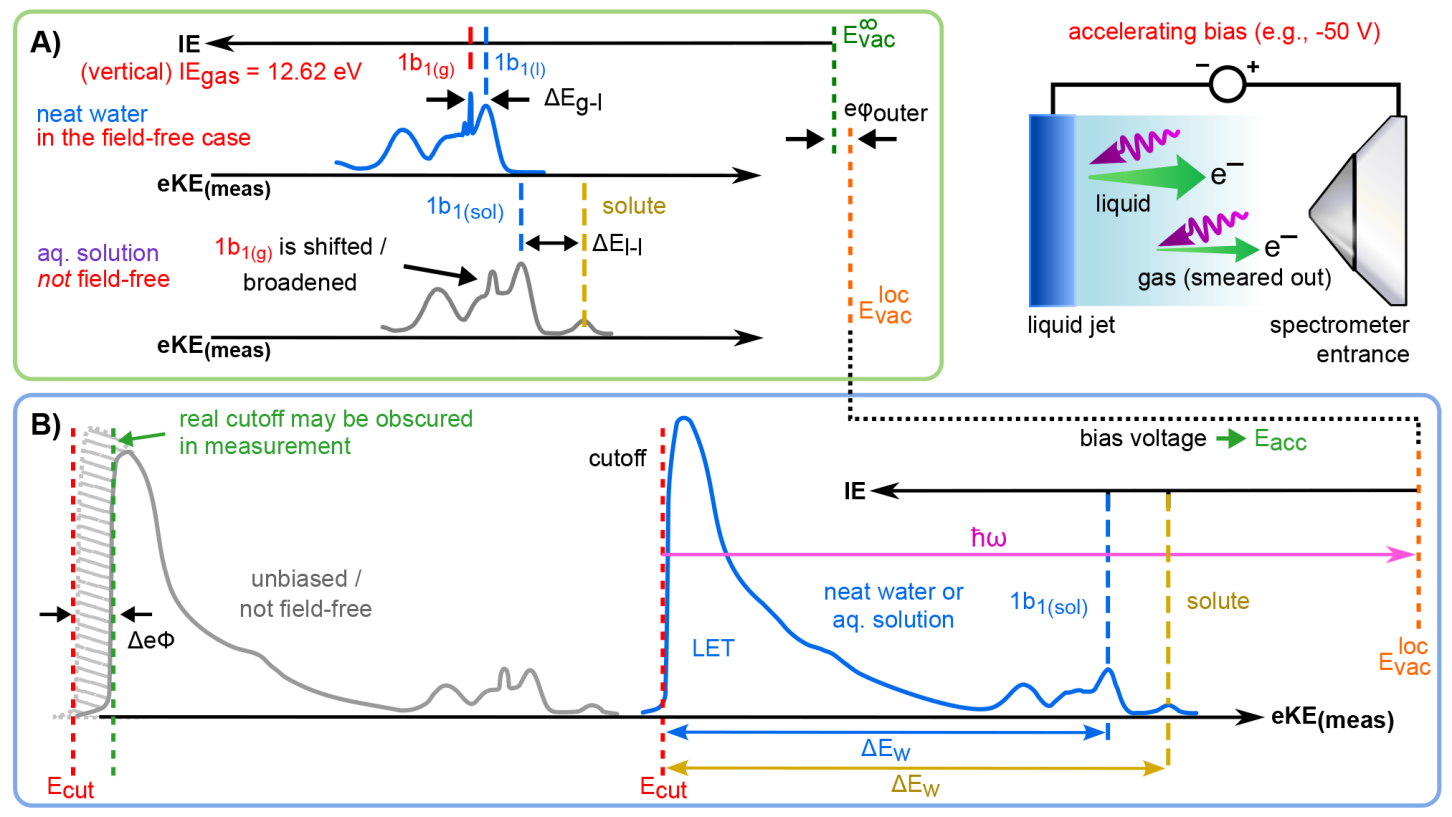
Schematics of the PE features used for energy-referencing. (A) Neat water (top) and arbitrary solution (bottom) PE spectra measured in an electrically grounded configuration. For the former, all potentials between the sample and analyzer are compensated, achieving “field-free” conditions. (B) Novel liquid-phase-energy-referencing scheme, where spectra are measured from a biased sample (see the experimental configuration sketch, top-right), producing the blue spectrum. The negative bias voltage shifts all liquid features to higher eKE, compared to the grounded case (gray), exposing the liquid-phase LET spectrum with the characteristic cutoff feature.

Electrons traveling through bulk matter have high elastic and inelastic collision probabilities. Scattering processes, including their eKE (and thus ℏω) dependence, have recently been detailed for liquid water.^3,14,23,24^ Briefly, native IE values can only be extracted from PEs that did not undergo inelastic scattering events. Condensed-phase inelastic electronic scattering processes (e.g., impact ionization) give rise to broad, additional signals that are well-separated (by >7 eV in the case of water) and isolable from the primary PE peaks. Still, PE features at higher IE often considerably overlap with this broad scattering background, complicating analysis. In addition, primary electrons that have lost energy in multiple inelastic scattering events, as well as electrons formed in impact-ionization cascades, accumulate in the low-energy tail, LET, which is ubiquitous in condensed-phase PE spectra (see, e.g., the spectrum in Figure 2B, detailed later). Electrons with the smallest resulting energies (quasizero eKE) give rise to the steep signal drop, leading to the “cutoff” edge at an energy, *E*_cut_, marking the minimum energy threshold for electron ejection into vacuum and, importantly, the zero point of the liquid-phase eKE scale.

In contrast to electronic scattering effects, low-momentum-transfer inelastic scattering, particularly so-called quasielastic scattering, contributes to the signal background in the energetic vicinity of a PE feature. The associated small-energy losses, most prominently related to vibrational excitations in water,^3^ can severely disturb native PES line shapes; in liquid water and aqueous solutions, this occurs for eKEs smaller than approximately 15 eV.^3^ The present Account deliberately focuses on IE and DE measurements with sufficiently high photon and eKEs (≥25 eV) to mitigate such detrimental quasielastic scattering effects. Furthermore, true elastic scattering inevitably reduces the anisotropy of (native) PE angular distributions,^25−27^ making it difficult to distinguish and quantify genuine gas-to-liquid-phase changes of electronic character caused by, e.g., the hydrogen-bonding environment.

Finally, upon reaching the liquid–vacuum interface, PEs must overcome any surface potential, which may be altered by surface dipoles. This solution-dependent interfacial potential is an intrinsic part of the overall ionization process and is manifested in all liquid-phase IE measurements. This, notably, guides us toward an alternative description of IEs, involving the Fermi level, *E*_F_, and eΦ.

## Ionization/Detachment Energies from Solutions: Vacuum Level Energy Referencing

In the previous section, we outlined the quantitatively different nature of gas- and liquid-phase photoemission. The differences largely arise from solution–component interactions and the surface associated with the latter. In water, these interactions largely occur via hydrogen bonds, which can be (and, so far, were predominantly) viewed as weak perturbations to the H_2_O molecular orbital structure and, hence, VIEs. The broader liquid-phase PES bands primarily result from the continuum of solvation-shell configurations, with somewhat different IEs. The larger eKEs (lower VIEs) measured in the liquid phase can be explained by solvent dielectric screening properties.^21,28^ Naturally, in the molecular-physics description, adopted in almost all previous aqueous-phase LJ-PES studies, the VIEs of liquid water were referenced to the vacuum level relevant for isolated (gaseous) species, namely, the vacuum level at infinity, *E*_vac_^∞^, referring to the transfer of a PE to an infinite distance from its origin. However, a different picture emerges for condensed-phase ionization. The appropriate vacuum level for liquid-phase ionization or detachment is the potential “just outside” the sample, sufficiently far away that the image charges disappear but close enough that the electron is still influenced by any surface dipole potential. This level is termed the local vacuum level, *E*_vac_^loc^, and is arguably the more relevant quantity for determining VIEs from condensed-phase samples, as it connects to the minimal energy input, i.e., ℏω, required to ionize a given bound state.

The difference between *E*_vac_^∞^ and *E*_vac_^loc^ is the (outer) surface potential, eφ_outer_, which in the case of neat liquid water may be caused by a small surface dipole; see the slight vacuum-level difference illustrated in Figure 2A. That is, an electron “just outside the surface” is generally still affected by the surface potential, and thus, the experienced “local” vacuum level usually differs from the theoretical *E*_vac_^∞^ level. Considering neat water, the net surface dipole potential is small—a few tens of millivolts,^29,30^ below common <100 meV LJ-PES uncertainty ranges—with the exact value yet to be experimentally determined.

As we will see, gas-phase VIE_vac_^∞^ values can be readily determined from measured eKEs, while corresponding liquid-phase values cannot, due to unknown surface potentials. However, even when ignoring such effects (for convenience), i.e., assuming VIE_vac_^∞^ = VIE_vac_^loc^, the measurement of VIE_vac_ is generally experimentally challenging due to the aforementioned parasitic potentials (e.g., surface charging). Let us correspondingly consider the valence PE spectrum presented in Figure 2A (blue, top trace). This was measured from a grounded, nearly-neat water LJ, with a small amount of electrolyte added to maintain conductivity, and ℏω = 40.813 eV (He II α line). Such an experiment inevitably measures electrons from both liquid and evaporated-gas-phase water molecules, where the gas-phase VIE is well-known.^31^ It may, therefore, be intuitive to obtain VIE_vac_ from the energy difference between the gas- and liquid-phase signals, Δ*E*_g-l_; Δ*E*_g-l_ is marked in Figure 2A to exemplify the separation of water’s lowest-IE, 1b_1_, PE peaks, VIE_1b1,gas_ – VIE_1b1,liq_. However, this approach is only valid if all parasitic potentials cancel to zero, such that there is no electric field between the LJ and electron analyzer, so-called “field-free” conditions. Otherwise, any parasitic potential will lead to an erroneous measurement of Δ*E*_g-l_.

Under favorable conditions, with a sufficiently large probing volume, sharp gas-phase PE signals can serve as an indication of zero field; i.e., no peak broadening occurs, as explained along with Figure 1B. For water LJs, attempts have been made to establish field-free conditions, particularly by properly compensating the streaming potential.^1,19^ However, it is easy to comprehend that such a gas-phase reference approach, determining Δ*E*_g-l_ (see Figure 2A), will inevitably fail when parasitic potentials cannot be nullified (or quantified). Indeed, for a given aqueous solution of arbitrary concentration, exhibiting a pronounced streaming and/or surface potential, this is presently unfeasible. As a consequence, PES spectra from aqueous solutions typically exhibit broadened and energy-shifted gaseous 1b_1_ PE peaks, and Δ*E*_g-l_ is arbitrary. This is illustrated by the gray, lower spectrum in Figure 2A. An associated fully objectionable practice, although a common practice in the EUV and soft X-ray LJ-PES community, is to assume that the neat-water VIE_vac,1b1_ energy is fixed and to use the energy gap between VIE_vac,1b1_ and a solute peak, Δ*E*_l-l_, to determine solute VIEs, neglecting any effect of solutes on water’s electronic structure and surface dipole potential.^32^

Considering the challenges outlined above, it becomes clear that the general determination of accurate VIE_vac_ values from liquids requires additional spectral information. An associated possibility is the aforementioned low-eKE cutoff energy, *E*_cut_, which can generally be shifted away from the difficult-to-measure near-zero-eKE region by applying a negative bias voltage to the LJ, as long as the solution is sufficiently electrically conductive to support the applied bias.^1^ The bias voltage, −*V*_bias_, is used to uniformly accelerate the PEs from the liquid sample toward the grounded detector (see the top-right of Figure 2), resulting in a rigid shift of the entire liquid-phase PE spectrum, including the cutoff feature, to higher eKEs. This is illustrated by the blue spectrum in Figure 2B. A valuable side effect is that, for sufficiently large −*V*_bias_, the gas-phase signal is broadened to a degree that it can be effectively suppressed; to a good approximation, a gas-phase-signal-free, liquid-phase PES spectrum can be obtained.^1^ After determining *E*_cut_ from the biased-jet PE spectrum, and together with a precisely known photon energy, VIE_vac_^loc^ can be precisely determined via VIE_vac_^loc^ = ℏω – (eKE_peak_ – *E*_cut_) for any solute or solvent PE feature of interest; note that neither the bias nor any other potentials appear in this equation because they are effectively canceled, making them irrelevant. For instance, VIE_vac,1b1_ = ℏω – Δ*E*_w,1b1_ for the liquid water 1b_1_ peak, from neat water or any aqueous solution, can be accurately and independently determined via the measured eKE separation between the 1b_1_ peak maximum and *E*_cut_, i.e., the spectral width, Δ*E*_w,1b1_ = eKE_1b1_ – *E*_cut_.

## Liquid Water and Aqueous Solution Electronic Structure

In discussing Figure 2, we outlined an experimental concept to accurately measure VIEs from neat water and aqueous solutions, neither obscured by jet-surface charging, relying on water (or alternative) gas-phase energies, nor referencing to the neat liquid water VIE_vac,1b1_. Prior to the implementation of these concepts, the neat water VIE_vac,1b1_ value was amended several times in the 20 years since the initial report: VIE = 10.92 eV,^4^ refined to VIE = 11.16 eV,^21^ and later 11.31 eV.^33^ Notably, all of these measurements were based on the gas-phase-reference approach, sketched in Figure 2A, with the aforementioned drawbacks. The precise value of VIE_vac,1b1_ = 11.33 ± 0.03 eV^1^ (and the respective VIE of any arbitrary aqueous solution, see below) can now be experimentally determined from the measurement of *E*_cut_ and the eKE of the solution PE feature, as explained with Figure 2B. This approach was applied to measure VIE_vac,1b1_ for a range of well-calibrated photon energies, spanning the (vacuum) ionization threshold up to more than 900 eV above it, as we recently reported in ref (1). This large variation in ℏω, associated with broad eKE and PE mean free path ranges,^25,34,35^ allowed the experimental probing depth to be varied between the surface and (predominantly) bulk-liquid regions. A small to negligible VIE depth-dependence was observed, implying that the combined effect of partial interfacial hydration^36^ and variable sampling of water’s surface dipole potential leads to a <50 meV change in liquid water’s VIE.

**Figure 3:**
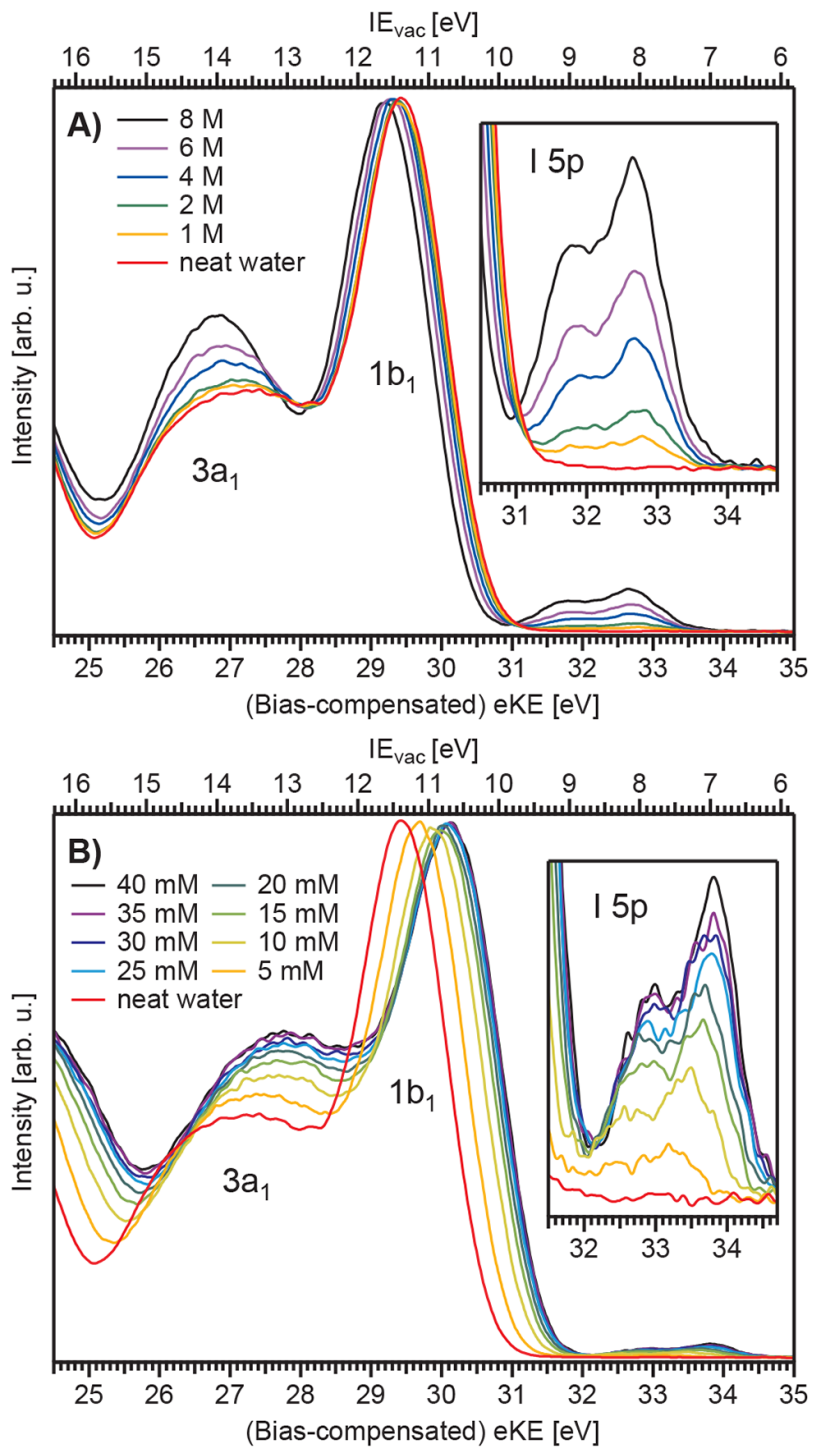
Series of valence-band spectra for aqueous solutions of different concentrations of (A) NaI and (B) tetra-*n*-butylammonium iodide (TBAI). All spectra are intensity normalized to the water 1b_1_ peak height. The red curve in each panel represents neat water, where NaI was added to 50 mM concentration to maintain conductivity. The panel insets show enlarged iodine-5p electron photodetachment features. For clarity, a ten-point binomial smoothing routine was applied to all spectra. The unsmoothed data plots and further details can be found in ref (2). Reproduced with permission from ref (2). Copyright 2022 Royal Society of Chemistry.

The same *E*_cut_-based measurement protocol that was used to obtain VIE_vac,1b1_ from neat water can be applied with similar accuracy to determine solvent and solute VIEs/VDEs from arbitrary solutions; again, see Figure 2B. Solute-induced changes of water’s electronic structure were initially explored with NaI aqueous solutions of varying concentration.^2,32^ Associated results are presented in Figure 3A, which shows 50-mM-to-8.0-M-concentration PE spectra from NaI-aqueous-solution microjets; the lowest concentration was implemented to maintain sufficient conductivity for PE experiments but is otherwise considered indistinguishable from neat water. Measurements were performed with a −25 V biased LJ, using a He II α plasma discharge source with ℏω = 40.813 eV. Here, only the outer-valence spectral regions are displayed, covering the water 3a_1_, 1b_1_, and spin–orbit-split iodide I^–^ 5p_3/2_/5p_1/2_ doublet peaks. The bias-corrected eKEs (where *E*_cut_ = 0 eV) are shown on the bottom axis, and the corresponding IEs/DEs, established using Δ*E*_w_ (as with neat water, see Figure 2B), are shown on the top axis; the signal intensities are normalized at the water 1b_1_ peaks. Respective LETs, with characteristic low-energy cutoffs, are not shown here but were previously presented in ref (2). With increasing NaI concentration, the water 1b_1_ peak shifts to larger VIE by ∼270 meV. This is accompanied by a significant change of the characteristic flat-top, water 3a_1_ peak shape. This results from a decreasing energy spacing between the 3a_1_ intermolecular bonding and antibonding components, due to weakened intermolecular electronic interactions between water units as they are replaced by ions at higher salt concentrations.^32^Figure 3A also reveals concentration-dependent, iodide-5p-solute-feature VDE shifts, enlarged in the inset. Quantitative analysis, applying appropriate peak fitting and modeling multilayer adsorption,^2^ reveals only a moderate-to-low surface enrichment of iodide, compensated by the Na^+^ counterion in the subsurface. Particularly, any differential segregation, implying the formation of an electric double layer (separating the anions and cations by approximately 3 Å),^37^ is argued to be counterbalanced, and a change in eΦ is, if occurring at all, very small. The ∼270 meV VIE_vac,1b1_ shift—despite the transition from essentially hydrogen-bonded neat liquid water to a crystalline-like liquid phase—can be rationalized as an isolation and stabilization of the nonbonding 1b_1_ electrons by the charge-dense sodium cation.^32^

## Solution Work Functions and the Fermi-Level Reference

**Figure 4:**
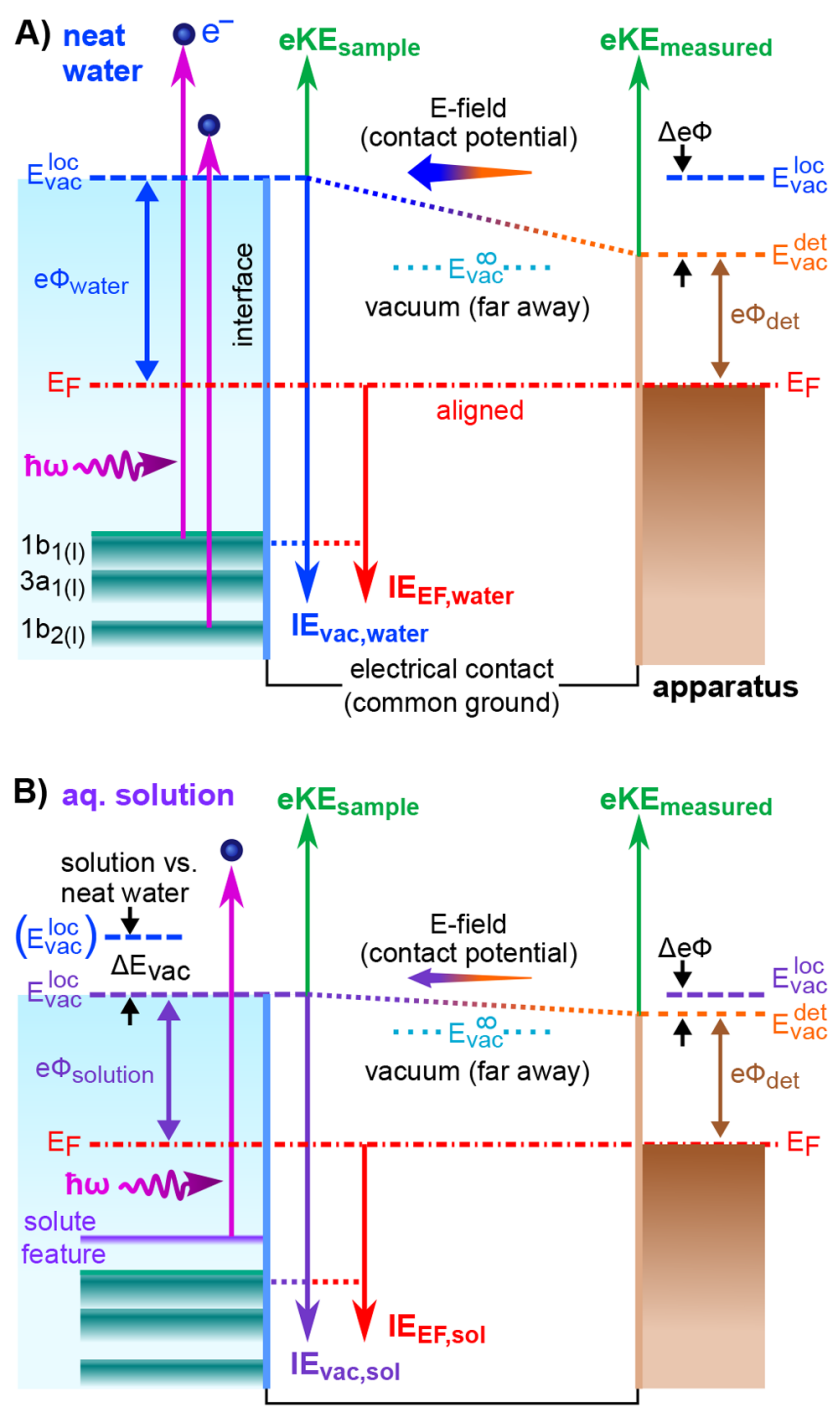
Basic energy scales and potentials relevant for valence-band PE spectroscopy, shown for (A) neat liquid water and (B) an exemplary aqueous solution.

Access to accurate VIE_vac_^loc^ values alone is still insufficient to access explicit liquid-surface properties, where in the present context, eΦ is of primary importance. Formally, eΦ is the minimum energy required to remove an electron nominally residing at *E*_F_, deep inside a material, and place it at rest “just outside” the surface, thus connecting to *E*_vac_^loc^. eΦ, *E*_F_, and *E*_vac_^loc^ are presented in the energy-level diagrams shown in Figure 4. Note that almost all liquids can be considered to be large-gap semiconductors, with *E*_F_ located within the band gap and an electronic density of states of zero at *E*_F_. How can we correspondingly determine *E*_F_ and eΦ from such systems?

We again consider two liquids, neat water and an aqueous solution, in good electric contact with the grounded apparatus, assuming that this corresponds to Fermi-level alignment between the bulk solution and detector. Figure 4 shows that eΦ can, in principle, be inferred from the energy difference between *E*_vac_^loc^ and *E*_F_ or, by proxy, IEs referenced to these two levels, i.e., the difference between IE_vac_ and IE_EF_. As eΦ is a property of the respective solution and IE_vac_ scales with eΦ, the difference between IE_vac_ and IE_EF_ changes when comparing different solutions (compare to Figure 4A,B). In the example shown in the figure, the solution eΦ is assumed to be smaller than that of neat liquid water.

While it is straightforward to measure VIE_vac_, as explained in the previous section, the challenge in determining eΦ lies in extracting VIE_EF_ from the experiment, as this quantity is inaccessible from the solution alone. The route to locate *E*_F_ of the apparatus is to simply measure the Fermi-edge spectrum from a metallic reference sample, grounded to the detector. However, special conditions must be met to associate this measured *E*_F_ position with a liquid of interest. For an arbitrary solution, the hardly quantifiable parasitic potentials affect the measured PE eKEs. Hence, separately determined *E*_F_ positions and affected-solution VIEs generally yield incorrect eΦ values, and data acquisition conditions must be designed that minimize all *undesirable* potentials. As shown in Figure 4A, *E*_F_ alignment between the solution and apparatus inevitably implies that the respective vacuum levels are not aligned, the energy difference being the contact potential difference (or Volta potential),^38^ ΔeΦ. We emphasize that proper *E*_F_-alignment *requires* the presence of ΔeΦ. This further implies that the to-be-established experimental conditions do *not* correspond to zero-electric-field conditions between the LJ and detector, as identified by associated sharp gas-phase peaks and typically occurring when all parasitic potentials are canceled by dissolving an empirically determined amount of salt. However, the conditions desired here require compensation of just the parasitic potentials, but not ΔeΦ. This cannot be gauged by the sharpness of the gas-phase peaks but rather requires elimination of only electrokinetic charging by adding an accurate amount of salt to the solution,^18,19^ neither over- nor undercompensating. For neat liquid water, this phenomenon is extensively studied and can be reasonably well accomplished, yet it is hardly achievable in the presence of solutes with arbitrary concentration, with some fortunate exceptions, as detailed below.

## Surface-Active Solutes: Solution Electronic Structure versus Work Function

We have seen that VIE_vac,1b1_ measurements alone are insufficient to determine solution eΦs. It is, therefore, instructive to consider the case of a high-surface-concentration surfactant in liquid water, where a significant surface-dipole buildup and related eΦ change is expected, along with a potential solvent electronic structure change. Both changes will affect VIE_vac_ and cannot be disentangled in the experiments discussed so far. However, consideration of *E*_F_ and Figure 4 will allow us to separate these effects. First, though, we will focus on VIE_vac_ results measured from the surface-active salt solution, analogous to the NaI aqueous solution data of Figure 3A.

Extended valence PES spectra recorded from 5 to 40 mM average-concentration aqueous tetra-*n*-butylammonium iodide (TBAI) solutions are presented in Figure 3B. Here, large (up to 0.7 eV) water VIE_vac,1b1_ shifts are observed, despite the low bulk solute concentration, with lower VIE values emerging with increasing concentration. Furthermore, the shifts are essentially saturated at ∼25 mM concentration, corresponding to approximate completion of a TBAI surface monolayer.^2,39^ One can correspondingly speculate that such VIE shifts are caused by a change of eΦ, where the decrease in eΦ would be consistent with a PE eKE increase, due to acceleration by a dipole field, implying a negative charge (I^–^) pointing into the solution and positive charge (TBA^+^) residing at the top surface. However, the quantitative distinction between eΦ and electronic structure changes requires direct experimental access to *E*_F_, necessitating further measurements from *grounded* LJs under special experimental conditions (see Figure 4).

**Figure 5:**
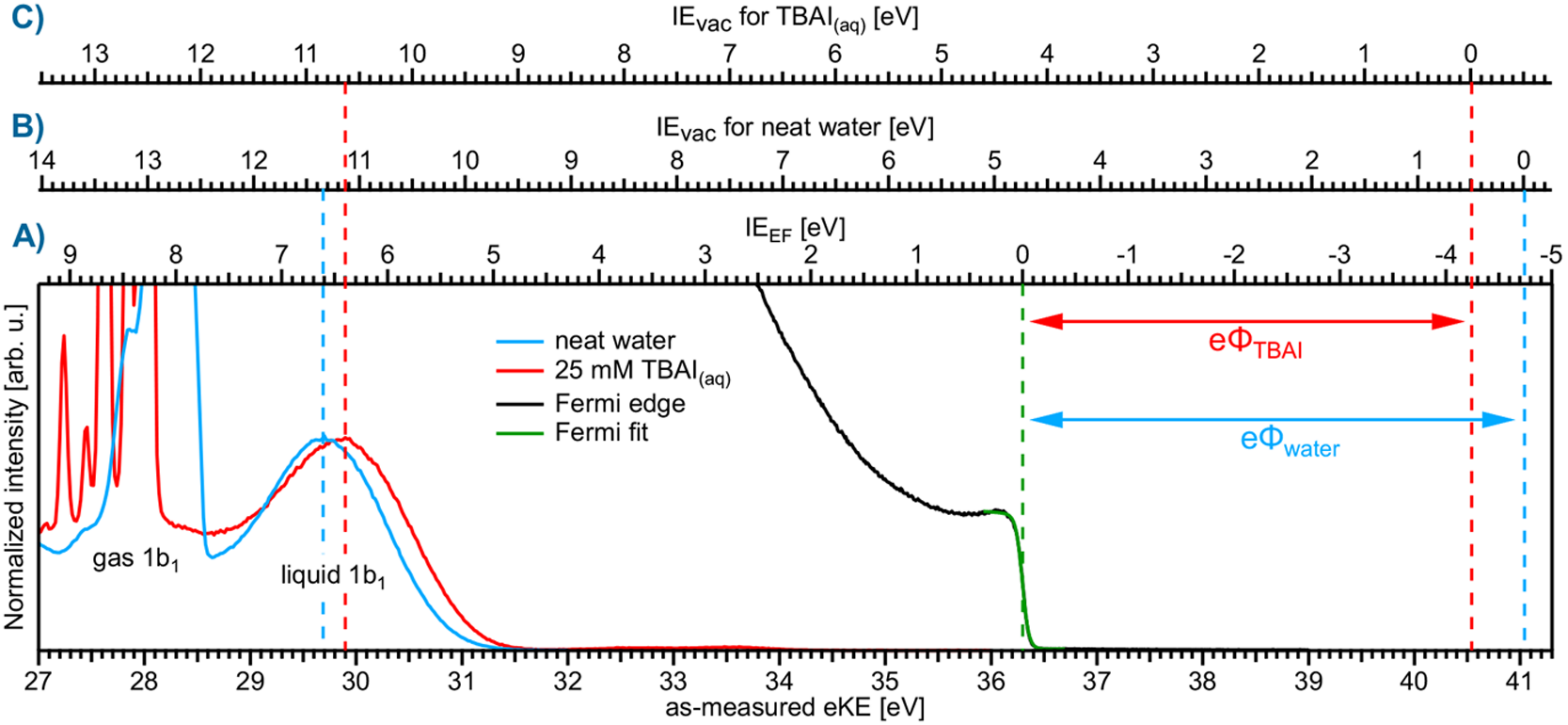
Valence PE spectra from neat liquid water (blue) and a 25 mM TBAI aqueous solution (red), both recorded from grounded LJs and measured at ℏω = 40.813 eV; the bottom axis shows the as-measured eKE scale of the detector. Measurements were performed under conditions free of any potential other than the contact potential, ΔeΦ (see Figure 3). The black curve shows the Fermi edge spectrum recorded from a reference metal, as fitted with a Fermi function (green line), the center position of which defines the zero-point of the VIE_EF_ energy scale (A). IE_vac_ axis for neat water (B) and for TBAI_(aq)_ (C), defined using the liquid 1b_1_ peak as an anchor point. The difference between VIE_EF_ and VIE_vac_ for each peak gives, per definition, the solution work function, eΦ. Reproduced with permission from refs (1 and 14). Copyright 2021 and 2022, respectively, Royal Society of Chemistry.

As mentioned above, parasitic potentials must be eliminated while maintaining ΔeΦ between the LJ and the detector, such that the correct *E*_F_ position—established from a metallic-reference-sample PES measurement—can be assumed within the liquid spectrum. This is exemplified for nearly-neat liquid water in Figure 5 (light blue spectrum), where NaCl was dissolved to 50 mM concentration to nullify Φ_str_, in accord with previous reports.^18,19^Figure 5 was produced from a LJ formed by a grounded platinum–iridium pinhole-disc, instead of a more-often-used glass nozzle, further mitigating streaming-potential issues. The separately measured apparatus *E*_F_ position is also shown, as a reference-metal PE spectrum recorded from the platinum–iridium, LJ-injection pinhole. As-measured eKEs are presented on the bottom axis, without any bias-voltage or associated eKE-scale correction. The neat-water-spectrum gas-phase peak is broadened, indicating the presence of a nonzero contact potential. Under these conditions, a direct relation to *E*_F_ is possible, and a VIE_EF_ reference axis (Figure 5A) can be established with VIE_EF_ = 0 eV at *E*_F_. This results in VIE_EF,1b1_ = 6.60 ± 0.08 eV for neat liquid water, and a direct comparison to VIE_vac,1b1_ yields, per definition, eΦ = VIE_vac,1b1_ – VIE_EF,1b1_ = 4.73 ± 0.09 eV (compare axes A and B in the figure).^1^ Unfortunately, however, Φ_str_ for an arbitrary solution is usually not zero, which generally yields erroneous results when attempting to refer to a fixed *E*_F_ position.

In the special case of TBAI_(aq)_, exemplified here by the 25-mM-TBAI-solution PE spectrum (Figure 5, red), Φ_str_ ≈ 0 V was found. We speculate that the TBA^+^–nozzle interaction is suppressed due to the carbon chains providing electrical screening or physical separation from the inner walls, thus preventing buildup of a charge separation layer, as will be discussed in a forthcoming publication. Furthermore, the bulk concentration of 25 mM was large enough (e.g., compared to NaI in Figure 3A) to ensure sufficient electrical conductivity, preventing ionization-induced sample charging. Notably, *E*_F_, the zero point of the VIE_EF_ scale, is the same for neat water and TBAI_(aq)_; i.e., we assume that only the contact potential is acting in both cases, so that a direct comparison can be made to the metal-reference spectrum. As shown, the positions of the water 1b_1_ peaks on the VIE_EF_ scale are inequivalent: 6.60 ± 0.08 eV for neat water and 6.45 ± 0.08 eV for 25 mM TBAI_(aq)_.^1^ This demonstrates that the aforementioned VIE_vac,1b1_ change is at least partly caused by a change to the solvent electronic structure, unrelated to eΦ (which is not part of VIE_EF_). The difference between VIE_vac,1b1_ and VIE_EF,1b1_ yields eΦ_TBAI_ = 4.25 ± 0.09 eV; i.e., the majority of the observed VIE_vac_ shift is still carried by the rather large TBAI-solution eΦ reduction (compared to 4.73 ± 0.09 eV for neat liquid water).^1^

In conclusion, under the right conditions, it is possible to disentangle solute-induced work-function and electronic-structure effects in liquids. However, the stringent constraints imposed on the sample, and the necessary assumptions about streaming and other parasitic potential mitigation, currently severely limit the applicability of this technique. It is correspondingly highly desirable to develop more direct methods of accessing *E*_F_ from aqueous solutions, a challenge that we are striving to meet within our laboratories.

## Conclusion

In this Account, we reviewed accurate PE spectra energy-referencing concepts for liquid water, aqueous solutions, and liquids in general. We introduced three fundamental potential levels for energy-referencing (vertical) ionization/detachment energies, (V)IEs/DEs, and discussed their applicability and shortcomings. First, the vacuum level at infinity, *E*_vac_^∞^, was presented, relevant for gas-phase spectroscopy. Second, an all-liquid-phase energy-referencing scheme was introduced, utilizing the low-energy spectral cutoff, *E*_cut_, to establish an absolute energy scale with respect to the local vacuum level just outside the liquid sample surface, *E*_vac_^loc^. This energy level is more relevant when discussing VIEs/VDEs of solutes and solvents in arbitrary solutions at arbitrary concentrations, as demonstrated for several aqueous salt solutions. The third energy reference is the Fermi level, *E*_F_, yielding an associated VIE_EF_ scale and potentially enabling access to solution work functions, eΦ. Currently, however, this requires favorable experimental conditions with good control of parasitic, especially liquid-streaming, potentials. Overall, the liquid-phase energy-referencing schemes described here represent novel and precise tools to study chemical properties, bonding environments, and structural changes in arbitrary solvents and solutions, both on absolute energy scales and with unprecedented fidelity.
